# DNA barcoding reveals polymorphism in the pygmy grasshopper *Tetrix bolivari* (Orthoptera, Tetrigidae)

**DOI:** 10.3897/zookeys.582.6301

**Published:** 2016-04-21

**Authors:** Ling Zhao, Li-Liang Lin, Zhe-Min Zheng

**Affiliations:** 1 College of Life Science, Mianyang Normal University, 166 West Mianxing Road, Mianyang 621000, China; 2 Institute of Zoology, Shaanxi Normal University, 199 South Chang’an Road, Xi’an 710062, China

**Keywords:** Crypsis, DNA barcoding, frequency, polymorphism, Tetrix bolivari

## Abstract

Many pygmy grasshopper species exhibit colour-marking polymorphism. However, this polymorphism in some species, such as *Tetrix bolivari*, is almost unknown. The aim of this work is to identify using DNA barcoding the colour-marking polymorphic morphs of this pygmy grasshopper species collected from both grass and sand microhabitats. Analysis by NJ clustering and pairwise distances indicated that all specimens collected showing colour-marking polymorphism are species of *Tetrix bolivari*. Haplotype network construction showed ten different haplotypes from a total of 57 *Tetrix bolivari* individuals with H1(82.5%) being the most common type and it also displayed low divergence within *Tetrix bolivari* population. The haplotype analyses were consistent with the NJ clustering. Our field census showed the frequency of *Tetrix bolivari* morphs differed significantly, with the rank order of morphs (from high to low) typeA_1_, type B_1_, type A_2_, type A_3_, type A_4_, type A_5_, type A_6_, type A_7_, type B_2_, type B_3_, and type B_4_. The most common type A morphs were without contrasting markings, while the rarer type B morphs have contrasting white markings. We suggest that type B morphs have greater camouflage effects against natural backgrounds such as grass or sand than type A morphs. Both our field census and haplotype analysis revealed that type A has higher frequency and more haplotypes than type B.

## Introduction

Pygmy grasshoppers are typical examples of polymorphic species (Holst 1986, Ichikawa et al. 2006). Different species show colour-marking polymorphism. Moreover, some species are highly polymorphic in colour and markings even within a single population (Forsman 2000). Such polymorphism has adaptive significance which can provide camouflage for the species against their natural backgrounds (crypsis), such as grass or sand (Ruxton et al. 2004, Stevens and Merilaita 2009, Cott 1940, Thayer 1909). The degree of camouflage differed among the colour-marking polymorphic morphs. Usually morphs with contrasting markings have greater camouflage against their natural backgrounds than those without them. However, the degree of camouflage of the morphs was not consistent with the morph frequency. It was reported the frequency of morphs with various types of markings differed significantly between the grass and sand microhabitats. Overall, morphs with contrasting markings were rarer in both microhabitats, although they were more cryptic (Kaori et al. 2010). The more cryptic morphs are not common in the microhabitats. Furthermore, for certain morphs with contrasting marking, such as longitudinal morphs of *Tetrix japonica*, they tended to be more common in the sand microhabitat where they were more conspicuous compared to the grass background where the markings provided a stronger camouflage effect for them. Therefore, the morph frequency cannot reflect the degree of crypsis.

Many pygmy grasshopper species exhibit discontinuous variation in colour and pattern of the pronotum, such as *Tetrix japonica* (Kaori et al. 2010), *Tetrix undulate* (Ahnesjo and Forsman 2003) and *Tetrix subulata* (Forsman 1997, 2000, 2001) and there is a strong tendency for the general patterns to be repeated in different genera and species (Nabours 1929, Fisher 1939). However, there are no publications on polymorphism in pygmy grasshoppers in China.

The Tetrigidae is an ancient group of Orthoptera with relatively uniform body structure. Most Tetrigidae are small, inconspicuous orthopterans about one cm long. They are terricolous and inhabit humid habitats, and some species are semi-aquatic (Podgornaya 1983, Paranjape et al. 1987).

In China, the Family Tetrigidae contains 15 genera, including the large genus *Tetrix*, which currently has 88 species (Deng et al. 2008). Generally, this Family of grasshoppers is among the least-studied groups of Orthoptera and most publications mainly focused on descriptions of new species and morphological taxonomy in China. Their ecology and biology are almost unknown.

*Tetrix bolivari* is one of the least studied species of China Tetrigidae but with wide distribution. Few published reports provide molecular data about its phylogeny (Fang et al. 2010, Chen and Jiang 2004, Jiang et al. 2002), and data on the activity, feeding biology and vibratory communication can be found in one study (Petr et al. 2011). Polymorphism within *Tetrix bolivari* adults has not been reported.

In this paper, we collected pygmy grasshoppers from both grass and sand microhabitats with large variation in body colouration and markings. To examine whether they are the same species with various colour-marking morphs or they are the different species, we conducted identification experiments using the protein-coding cytochrome c oxidase subunit I (COI) region as a DNA barcode. In addition, we conducted a field census of the morphs in the microhabitats (sand and grass) to confirm the grasshopper morph frequency.

## Materials and methods

### Sampling

Adult pygmy grasshoppers were collected from both grass and sand microhabitats in Mianyang, Sichuan Province, China in July to August, 2013. All morphs are characterized by both a long pronotum that extends beyond the apex of the abdomen and highly reduced forewings. They are small (males, 11.8–16.0 mm; females, 13.5–17.0 mm) and exhibit extraordinary variation in the colour and markings of the pronotum from black, through yellowish-brown to light grey or white, with some individuals being monochrome and others having spots, markings or distinct patterns on the pronotum (and also on the hind legs) such as a narrow light yellowish longitudinal stripe on the mid-line of the upper surface of the pronotum or whitish and blackish markings on the dorsal surface of the pronotum.

Fifty-seven specimens of different colour-marking morphs were preserved in 100% ethanol and stored at –4 °C for identification experiments.

### Identification experiments

DNA extraction: DNA from the tissues of the grasshoppers was extracted from the hind leg using a routine phenol/chloroform method (Zhou et al. 2007).

PCR amplification and sequencing: The DNA was ampliﬁed using polymerase chain reaction (PCR) in an ABI thermocycler. The following primers were used for amplication of the COI gene: 5’-TYTCAACAAAYCAYAARGATATTGG-3’ and 5’-T AAACTTCWGGRTGWCCAAARAA TCA-3’. PCR reaction was carried out in a total volume of 15 ul containing 1ul DNA template, 7.5 ul Mix (2×Taq DNA Polymerase, 2×PCR Buffer, 2×dNTP), 1 ul of each primer and 4.5 ul PCR-grade RNase-free water. Thermo-cycling conditions were as follows: one initial cycle of 4 min at 94 °C followed by 35 cycles of 94 °C for 15 s, 46 °C for 20 s, 70 °C for 90 s, with ﬁnal step of 72 °C for 7 min. The PCR products were visualized on 1% agarose gels and then sequenced with both the forward and reverse primers by Shanghai SANGON after separation and purification.

Data analysis: The 57 sequences were aligned using CLUSTALX and the standard 658bp was kept for the following analysis (GenBank Accession nos KU570134-KU570190). The morphological identiﬁcation for one individual (No. 57) suggested it was *Tetrix bolivari* (Tetrigidae, Tetriginiae, *Tetrix*). The 56 remaining individuals plus the individual *Tetrix bolivari* were analyzed together in NJ analysis by MEGA version 5.0, together with another 4 *Tetrix* species, 1 *Alulatettix yunnanensis* and *Teleogryllus emma* as outgroup.

The Kimura 2-parameter (K2P) model of base substitution was used to calculate pairwise genetic distance in MEGA 5 software. Species discrimination in DNA barcoding studies also depends on establishment of threshold interspecies nucleotide divergence. In this study, nucleotide divergence of 3% was considered as a threshold between species as observed in Orthopterans (Mao 2011).

The haplotype network based on 658 base pairs of COI sequences was constructed using the median-joining algorithm (Bandelt et al. 1999) implemented in NETWORK 4.1 (Forster et al. 2000).

### Field survey of the frequency of grasshopper morphs

Grasshoppers were collected using random sweeps with an insect net in both microhabitats and we counted the different colour-marking morphs in the field. Only adults were used in this research.

## Results

### Identification by DNA barcoding

**Figure 1.**
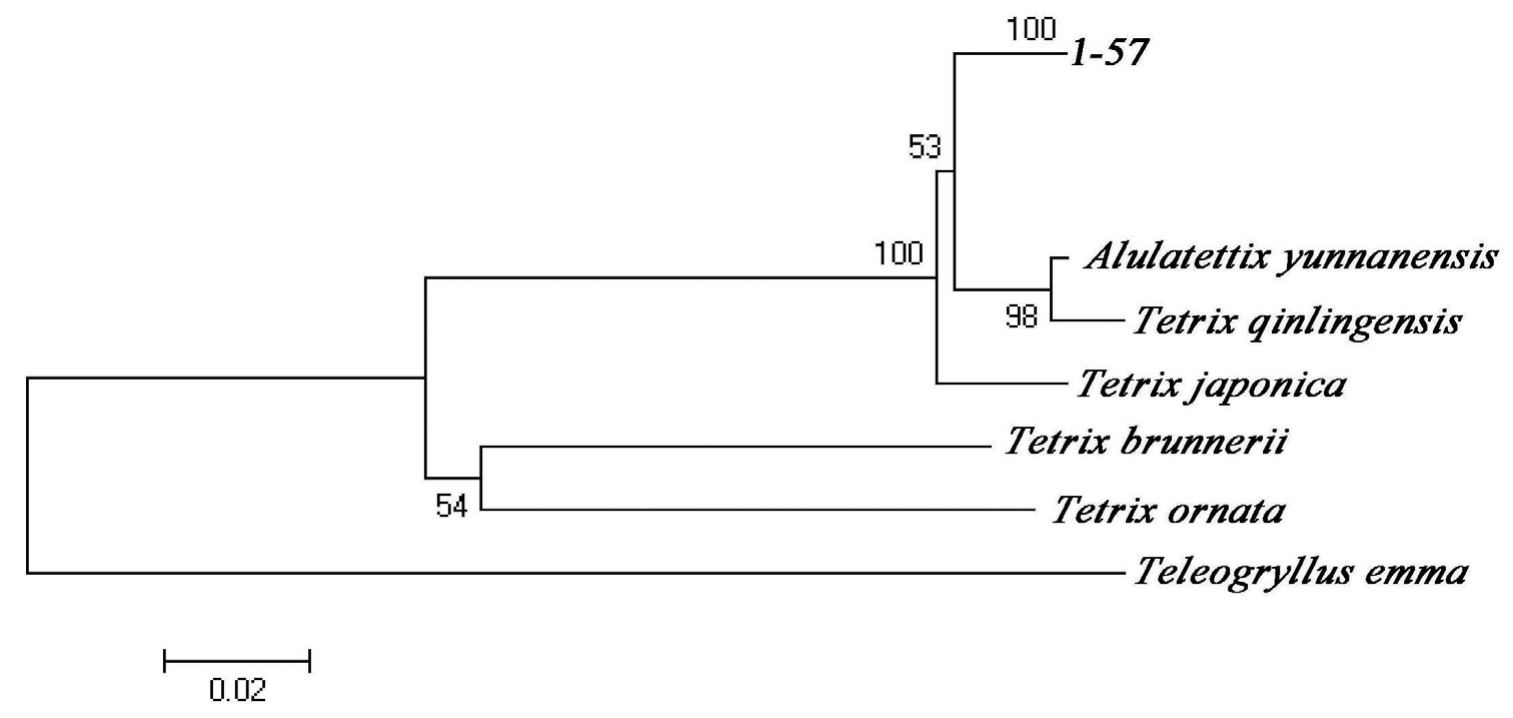
NJ clustering analysis of 62 sequences of pygmy grasshoppers using MEGA5 and K2P model. *Teleogryllus emma* is used as an outgroup. The clade 1-57 represents samples from this study.

Our NJ analysis showed that the 56 individuals with different colour-marking morphs clustered with *Tetrix bolivari* into one clade with bootstrap value of 100% (Fig. 1). The pairwise distances indicated the nucleotide divergence varied from 0.0% to 0.6% and the overall mean distance was 0.1%, which was far less than the threshold of 3% used for species discrimination (Suppl. material 1). According to the above analysis, we inferred that all pygmy grasshoppers with different morph types in this research are all species of *Tetrix bolivari*. Meanwhile, the NJ analysis showed that *Tetrix bolivari*, *Tetrix qinlingensis*, *Alulatettix yunnanensis* and *Tetrix japonica* have close relationship with a bootstrap value of 100%. However, another two species *Tetrix ornata* and *Tetrix brunnerii* were separated from the other species by interspecies nucleotide divergence ranging from 16.1% to 18%.

### Haplotype network construction

**Figure 2.**
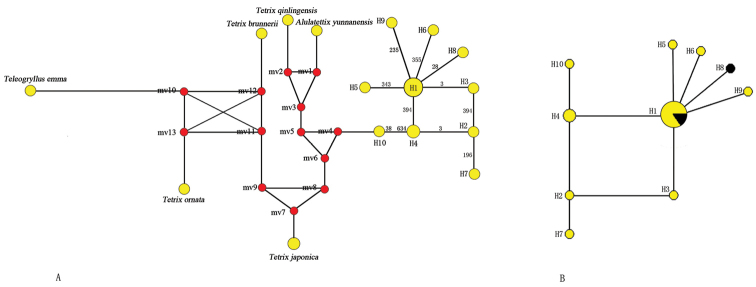
**A** Haplotype network from 63 sequences, including 57 *Tetrix bolivari* individuals, 4 *Tetrix* species, 1 *Alulatettix yunnanensis* and 1 outgroup (*Teleogryllus emma*) **B** Haplotype network of 57 *Tetrix bolivari* individuals combined with the morph types. Circle size is proportional to haplotype frequency. Lines drawn between haplotypes represent mutation events identified by the numbers corresponding to the positions at which the mutations were observed. Red points represent hypothetical haplotypes (median vector). Colours in **B** represent morph types. Yellow areas represent type A and black areas represent type B.

The haplotype analyses based on the 62 sequences were consistent with the NJ clustering (Fig. 2A). Ten different haplotypes were found in *Tetrix bolivari* population (Fig. 2). The most frequent haplotype was H1, occurring in 82.5% of individuals sampled (Table 1), indicating a low degree of variation in this population. This type was followed by H4 with much lower frequency (3.5%) and the remaining haplotypes occurred at a frequency of 1.8% (Table 1). Haplotypes H3, H4, H5, H6, H8 and H9 were each linked to H1 by a single nucleotide substitution at positions 3, 394, 343, 355, 28 and 235, respectively (Fig. 2A). The haplotype H7 was the most distant haplotype. It was separated from the haplotype H1 by 3 mutational events (Fig. 2).

**Table 1.** Frequency of the ten different haplotypes based on the 658 bp COI region in 57 *Tetrix bolivari* individuals used in this study.

Haplotype	Sequence code	Frequency (%)	Morph type	
			A	B
H1	1 3 4 5 6 7 8 9 11 12 13 14 15 16 19 21 22 23 24 25 26 28 29 30 31 32 34 35 36 37 38 39 40 41 42 44 45 46 47 48 49 50 51 53 54 56 57	47 (82.5)	40	7
H2	33	1 (1.8)	1	
H3	43	1 (1.8)	1	
H4	2 18	2 (3.5)	2	
H5	55	1 (1.8)	1	
H6	10	1 (1.8)	1	
H7	20	1 (1.8)	1	
H8	17	1 (1.8)		1
H9	27	1 (1.8)	1	
H10	52	1 (1.8)	1	
Total	57		49	8

### Frequency of different colour-marking morphs in the grass and sand microhabitats

**Figure 3.**
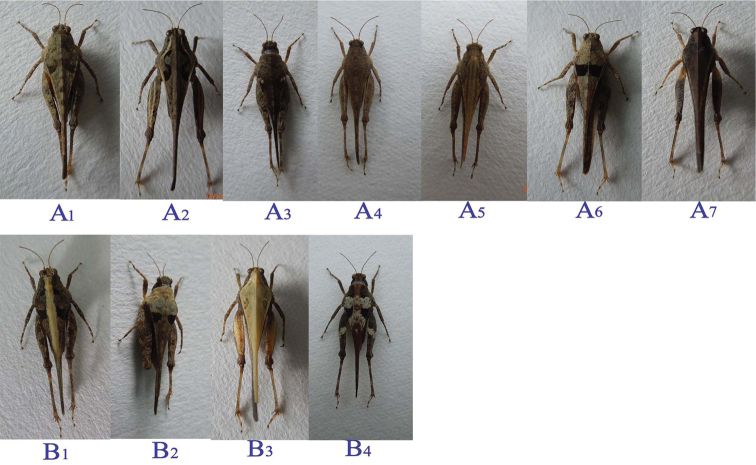
Morphs of pygmy grasshoppers (*Tetrix bolivari*) classified by type of colour and markings on the pronotum. A_1-7_ belongs to type A; B_1-4_ belongs to type B.

A total of 343 pygmy grasshoppers (*Tetrix bolivari*) were collected from both microhabitats and we categorized these morphs into 2 types (type A and type B) and 11 subtypes (type A_1-7_ and type B_1-4_) based on the colour and markings on the pronotum (Fig. 3). Type A generally has dark colours, such as black, brown and grey, without contrasting markings. While type B had white on the pronotum, mixed with the black markings. The number of each subtype is shown in Table 2. From this table, we can see that type A was dominant in the habitat with type A_1_ more common than other subtypes (type A, 79.3%; type A_1_, 29.7%), whereas type B was rare, especially type B_4_ which has obvious contrasting markings (type B, 20.7%; type B_4_, 0.9%).

**Table 2.** Morph types of pygmy grasshoppers (*Tetrix bolivari*) classified by the colour and markings on the pronotum.

Type	Number	Percentage
A_1_	102	29.7
A_2_	34	9.9
A_3_	31	9.0
A_4_	31	9.0
A_5_	28	8.2
A_6_	25	7.3
A_7_	21	6.1
A_1-7_	272	79.3
B_1_	43	12.5
B_2_	15	4.4
B_3_	10	2.9
B_4_	3	0.9
B_1-4_	71	20.7

In this study, 57 *Tetrix bolivari* individuals were used in NJ clustering and haplotype analysis, including 49 type A morphs and 8 type B morphs (Table 1). These type A morphs have nine different haplotypes and the 8 type B morphs have two haplotypes (Fig. 2B). Both morph types have haplotype H1 and it is the most prevalent type in both type A and type B.

## Discussion and conclusion

The aim of this study was to identify morphs of *Tetrix bolivari* using DNA barcoding and examine polymorphism and morph frequency in *Tetrix bolivari*. Both the NJ clustering analysis and pairwise distances indicated that all specimens in this experiment are species of *Tetrix bolivari*, which exhibits polymorphism in colour-marking morphs. Furthermore, we found the non-marked morph, spotted morph, and horizontal morph in *Tetrix japonica* also appeared in *Tetrix bolivari*. It was reported *Tetrix bolivari* is commonly found with *Tetrix subulata* (Adamovic 1969, Sardet 2007). Therefore, we suspect these two species presumably share some morph types, although we didn’t find any *Tetrix subulata* in our collection.

Our NJ analysis showed that *Alulatettix* was closely related to the three *Tetrix* species. Previous molecular studies (Fang et al. 2010) correspond with results of our research. From the morphological observations, *Alulatettix* and *Tetrix* belong to the subfamily Tetriginae, and both of these genera are similar in the head not projecting above upper level of pronotumand and the posterior margin of lateral lobes in lateral view with two concavities, but they differ in the shape of pronotum and degree of development of tegmina and hind wings. However, another two *Tetrix* species (*Tetrix ornata and Tetrix brunnerii*) were less close to the other species and showed higher interspecies nucleotide divergence, from 16.1% to 18%. Here, sequences of *Tetrix ornata* and *Tetrix brunnerii* downloaded from GenBank were submitted by the Biodiversity Institute of Ontario, Canada, a place far from China, while the other 2 *Tetrix* species (*Tetrix japonica* and *Tetrix qinlingensis*) and *Alulatettix yunnanensis* were contributed by Nanjing Normal University, China and *Tetrix bolivari* was from our lab. So, we inferred that the higher nucleotide divergence between *Tetrix ornata*, *Tetrix brunnerii* and other pygmy grasshopper species likely reflects geographical distribution differences. In addition, the molecular phylogeny also revealed that the genus *Tetrix* is not monophyletic (Fang et al. 2010, Chen and Jiang 2004).

Furthermore, a total of ten different haplotypes were detected in this single colour-marking polymorphic population with H1 (82.5%) being the most common type. The haplotype network displays a shallow divergence in this *Tetrix bolivari* population with a maximum of 4 base changes between the most divergent haplotypes. The haplotype analysis combined with the morph types showed type A has more haplotypes than type B and both of them have the prevalent haplotype H1.

Our field census of the polymorphism in the microhabitats (sand and grass) demonstrated that the different morphs of *Tetrix bolivari* were not equivalent in the frequency, with the rank order of morphs (from high to low) being typeA_1_, type B_1_, type A_2_, type A_3_, type A_4_, type A_5_, type A_6_, type A_7_, type B_2_, type B_3_, type B_4_. Generally, type A was more common than type B. Earlier work on *Tetrix japonica* has revealed the more common morphs, usually without contrasting markings, are not more cryptic in either grass or sand microhabitat. In contrast, the more cryptic morphs which have contrasting markings were rarer in each microhabitat (Kaori et al. 2010). Our field survey also showed that the more common type A morphs usually exhibited non-marked and mono-coloured basal colouration or any number of spots on mono-coloured basal colouration, while the rarer type B morphs had contrasting white markings. So, we infer that type B morphs have a much greater degree of crypsis against the natural backgrounds, such as grass or sand than type A morphs. There is no positive association between morph frequency and the degree of crypsis, which can be explained by the differential crypsis hypothesis (Forsman 1998).

## Supplementary materials

Supplementary material 1 Pairwise genetic distance (K2P) based on COI sequence using MEGA 5 Data type: specimens data This dataset is made available under the Open Database License (http://opendatacommons.org/licenses/odbl/1.0/). The Open Database License (ODbL) is a license agreement intended to allow users to freely share, modify, and use this Dataset while maintaining this same freedom for others, provided that the original source and author(s) are credited.Ling Zhao, Li-Liang Lin, Zhe-Min Zheng
